# Proceedings of the DeWitt County Medical Society

**Published:** 1857-05

**Authors:** W. W. Adams, C. Goodbrake

**Affiliations:** President; Secretary


*Proceedings of the De Witt County Medical Society.*

The Society met in annual session at the Court House, in Clinton, April 6th, 1857. The President, Dr. C. Goodbrake, in the chair.

On motion of Dr. Z. H. Madden, Dr. Harrison Noble, of Randolph's Grove, and Dr. Thos. P. Rogers, of Bloomington, who were present, were invited to participate in the proceedings of the meeting.

The Censors having reported favorably, on the application of John McHugh, M.D. he was, on motion of Dr. Edmiston, duly elected a member of this Society.

On motion, the Society then proceeded to the election of Officers for the ensuing year, which resulted as follows:—

Dr. Wm. W. Adams, *President*.

Dr. Evan Richards, *Vice-President*.

Dr. Chr. Goodbrake, *Secretary.*

Dr. Thomas K. Edmiston, *Treasurer.*

Dr. John Wright, Dr. E. Richards, Dr. Z. H. Madden, *Censors.*

Dr. Z. H. Madden and Dr. Christopher Goodbrake were elected delegates to the Illinois State Medical Society.

Dr. John McHugh, and Dr. B. S. Lewis were elected delegates to American Medical Association.

The retiring President then delivered his valedictory, which was listened to with marked attention by the members of the Society, and some of the citizens who had collected in the Court Room, in accordance with a public invitation.

Dr. W. W. Adams, President elect, then took the chair, and made a few very appropriate remarks, which were well received by the Society.

On motion of Dr. John McHugh, the following resolution was unanimously adopted:—

*Resolved,* That the thanks of this Society are due, and are hereby tendered, to Dr. Christopher Goodbrake, for his very able valedictory address. That the Doctor be requested to furnish a copy of his address; and that the Secretary be ordered to send it to the Editor of the *North-Western Medical and Surgical Journal,* with the request to publish the same.

On motion of Dr. Edmiston, Dr. Robert H. Brown, formerly a member of this Society, but now a citizen of Middletown Champaign County, was unanimously elected an Honorary Member of this Society.

The President appointed Dr. Z. H. Madden and Dr. John McHugh to write each an essay to be read before the Society at its next meeting.

Drs. Wright, McHugh and Goodbrake, were appointed a Committee on Publication.

Drs. Madden, Richards and Edmiston, were appointed a Committee on Prize Essays.

On motion of Dr. Madden, the following resolution was adopted :—

*Resolved,* That, hereafter the members of this Society will consider their fees due as soon as services are rendered. This resolution not to interfere with the right of the physician to extend the time for payment or settlement as long as he may deem proper.

The reading of essays being in order, Dr. Thos. K. Edmiston read an ably-written essay on the 'Pathology and Treatment of Typhoid Fever.' The essay called forth quite an interesting debate, in which Drs. Noble, Rogers, Richards, McHugh, Edmiston, and Goodbrake participated.

During the debate, the merits of nitric acid and strychnia, in some of the last stages of the disease, were discussed.

Dr. Noble gave the history of a very peculiar case of typhoid fever, which came under his treatment. The patient was a man about fifty years old, of good constitution, did not get very *sick* at any one time for the first two weeks. The pulse, *during the whole period of the disease*, remaining below the healthy standard. At no one time did it exceed 60 beats in the minute; most of the time only 50 beats per minute. The Dr. had known the patient well for several years—his pulse when in health beats 75 to 80 in a minute. The patient felt 'rather indisposed than sick'—not sick enough to keep his bed; told the Dr. that if it were not for his *indisposition* to move, he thought he would be able to go about the farm. This state of things lasted two weeks, when the Dr. was suddenly summoned to attend his patient; found him laboring under a profuse passive hemorrhage from the bowels—the patient sinking rapidly. The Dr. treated the case with large doses of dilut. nitric acid, which had the happy effect of saving his patient.

Dr. N. stated that he had found the nitric acid, in connection with turpentine, to be a very effectual remedy to arrest passive hemorrhage from the bowels, he having administered it in several almost hopeless cases, with very happy results.

Dr. T. P. Rogers also expressed great confidence in nitric acid to arrest hemorrhage occurring in the advanced stages of typhoid fever.

Dr. Edmiston combined the nitric acid with strychnia, in the following proportions—Strychnia gr. j. Nitric Acid ʒj. Tr. Opii ʒij. Aqua ℥ij. He thought that with this mixture and the turpentine emulsion, he had saved several patients who would otherwise have inevitably died. The Dr. mentioned several cases to illustrate the grounds upon which he made up his opinion.

Dr. McHugh wished to know the *modus operandi* of nitric acid and strychnia, in cases such as had been referred to. He was in the habit of treating such cases with sugar of lead and opium, with good success.

Dr. Goodbrake gave his views of the pathological condition, in the advanced stages of typhoid fever, and could very readily perceive why it was that the administration of nitric acid or the strychnine mixture should be attended with great benefit in the cases referred to by the gentlemen.

The discussion was continued until late in the evening, when,

On motion of Dr. McHugh, it was ordered that the proceedings of this meeting be published in the *North-Western Medical and Surgical Journal*, and

On motion of Dr. Richards, the Society adjourned to meet in quarterly session at Wapello, on the first Monday in July next, at 10 o'clock, A.M.

W. W. ADAMS, *President*.

C. Goodbrake, *Secretary*.

